# Incorporation of Chloramphenicol Loaded Hydroxyapatite Nanoparticles into Polylactide

**DOI:** 10.3390/ijms20205056

**Published:** 2019-10-11

**Authors:** Manuel Rivas, Marc Pelechà, Lourdes Franco, Pau Turon, Carlos Alemán, Luis J. del Valle, Jordi Puiggalí

**Affiliations:** 1 Chemical Engineering Department, Escola d’Enginyeria de Barcelona Est-EEBE, Universitat Politècnica de Catalunya, Av. Eduard Maristany 10-14, Ed I-2, 08019 Barcelona, Spain; manolorivas68@yahoo.es (M.R.); marcpele16@gmail.com (M.P.); lourdes.franco@upc.edu (L.F.); 2 Barcelona Research Center for Multiscale Science and Engineering, Universitat Politècnica de Catalunya, Av. Eduard Maristany 10-14, 08019 Barcelona, Spain; 3 B.Braun Surgical, S.A., Carretera de Terrassa 121, 08191 Rubí (Barcelona), Spain; pau.turon@bbraun.com

**Keywords:** hydroxyapatite, chloramphenicol, polylactide, electrospun scaffolds, drug encapsulation, drug release

## Abstract

Chloramphenicol (CAM) has been encapsulated into hydroxyapatite nanoparticles displaying different morphologies and crystallinities. The process was based on typical precipitation of solutions containing phosphate and calcium ions and the addition of CAM once the hydroxyapatite nuclei were formed. This procedure favored a disposition of the drug into the bulk parts of the nanoparticles and led to a fast release in aqueous media. Clear antibacterial activity was derived, being slightly higher for the amorphous samples due to their higher encapsulation efficiency. Polylactide (PLA) microfibers incorporating CAM encapsulated in hydroxyapatite nanoparticles were prepared by the electrospinning technique and under optimized conditions. Drug release experiments demonstrated that only a small percentage of the loaded CAM could be delivered to an aqueous PBS medium. This amount was enough to render an immediate bacteriostatic effect without causing a cytotoxic effect on osteoblast-like, fibroblasts, and epithelial cells. Therefore, the prepared scaffolds were able to retain CAM-loaded nanoparticles, being a reservoir that should allow a prolonged release depending on the polymer degradation rate. The studied system may have promising applications for the treatment of cancer since CAM has been proposed as a new antitumor drug.

## 1. Introduction

Chloramphenicol (CAM) is a powerful and efficient broad-spectrum antibiotic that has been used since 1947. It was isolated from a strain of *Streptomyces venezuelae*, although it is currently obtained in a synthetic manner. CAM is considered as bactericide drug but can act as a bacteriostatic at high concentrations or against very sensitive organisms. For example, CAM has been revealed highly effective against *Haemophilus influenzae*, *Streptococcus pneumoniae,* and *Neisseria meningitidis*, which are the three main bacteria responsible for meningitis [1]. Nevertheless, several side effects (e.g., urotoxicity and hematologic disorders) have been reported for CAM, making it somehow limited in its clinical application. Therefore, different strategies to get derivatives with improved pharmacological properties have been developed.

**Figure 1:**
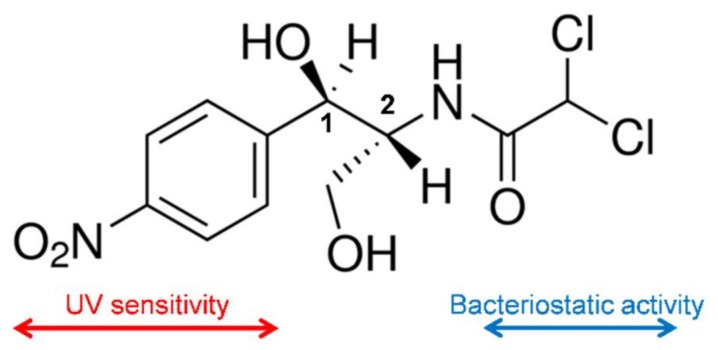
Chemical structure of chloramphenicol (CAM).

CAM is a white crystalline material, soluble in ethanol and relatively insoluble in water. The compound has two chiral centers (Figure 1), being the D-erythro isomer the one that has the main bacteriostatic activity (98%).

The mechanism of action of CAM consists in the interference of the bacterial protein synthesis, mainly by competitive inhibition of the peptidyl transferase activity of the bacterial ribosome. The dichloroacetyl moiety of CAM becomes essential for the antibacterial activity since it is similar to the aminoacyl-tail of tRNA [2]. Two binding sites for CAM in the 50S ribosome subunit have been determined from equilibrium studies [3], with affinity constants equal to 2 μΜ and 200 μΜ. Non-competitive type of inhibition mechanism has also been found depending on factors such as the nature of substrate, ionic buffer, or inhibitor concentration [4,5,6]. Clinical uses of CAM are, however, limited due to the development of a typical antibiotic-resistance through targeted mutations [7] and specific problems related to hematological disorders such as aplastic anemia [8].

Energy for eukaryotic cellular functions is provided by mitochondria through its capability to convert glucose into ATP. Normal human cells process glucose under aerobic conditions, firstly via a glycolysis step and subsequently via an oxidative phosphorylation step in the mitochondria that rendered pyruvate and CO_2_, respectively. Glucose metabolism is reprogramed by cancer cells, with the glycolysis process enhanced with respect to oxidative phosphorylation one (as typical of anaerobic conditions for normal cells) and the final energy production consequently modified according to named anaerobic glycolysis [9,10].

Nowadays, it has been postulated that efficient treatment of cancer can be performed by interfering with the mitochondrial biogenesis since stem cancer cells are strongly anabolic and consequently requires great activity for their survival and proliferative expansion. At this point, it is very interesting to note that mitochondria were originated from prokaryote organisms (i.e., endosymbiosis hypothesis) [11]. Thus, the inhibitory effects of antibiotics against bacteria would lead also to negative effects on mitochondrial protein synthesis and logically on the mitochondrial biogenesis [12,13]. The new strategy has been revealed effective for treatment of cancer and specifically different works concerned the use of CAM alone or in combination with anticancer drugs [13,14,15].

Hydroxyapatite (HAp) is a bioceramic material defined by the chemical formula Ca_10_(PO_4_)_6_(OH)_2_. This material forms part of human bones, tendons, and teeth. HAp is nowadays usually employed to prepare bionanocomposites due to its excellent biocompatibility, bioactivity, and osteoconductivity [16]. A great diversity of methods has been postulated to prepare HAp, but in general, those most employed are based on simple chemical precipitation [17,18]. This could lead to amorphous (ACP) or crystalline (cHAp) particles after subsequent aging and hydrothermal processes, respectively. Moreover, the morphology and size of particles can also be easily controlled by varying synthesis conditions. HAp particles have great received interest as carriers for drug delivery systems due to the facility to control texture, porosity, surface area, and even to allow their surface functionalization. In fact, HAp has been employed for the release of a large variety of therapeutic agents [19,20,21]. Thus, ACP and cHAp are calcium phosphates that differ in their crystallinity and have similar composition and Ca/P ratio. Namely, ACP is practically amorphous, being by extension this denomination used for particles with reduced crystallinity. In any case, cHAp is the most stable and less soluble calcium orthophosphate [16,18].

Probably, one of the major limitations concerning the effectiveness of anticancer drugs is related to the capacity of cells to expel these drugs from their interior through molecular pumps such as P-glycoprotein and the multidrug resistance-associated protein [22]. Chemotherapy efficiency is consequently reduced, being alternatively considered the incorporation of encapsulated drugs in order to avoid the failure of the treatment [23,24]. Calcium phosphate nanoparticles may be promising for the encapsulation of CAM, making it possible to achieve the following goals: (a) Effective incorporation into tumor cells via endocytosis, (b) evade being degraded within the lysosomal, (c) capacity to release CAM in the cytoplasm due to its acidic pH, and (d) capacity to display a CAM release rate sufficiently low to avoid activation of typical ejection pumps.

The present work is focused on the study of CAM encapsulation in calcium phosphate nanoparticles with different crystallinity (i.e., ACP and cHAp) and the evaluation of the corresponding release. Furthermore, the use of a biodegradable and biocompatible scaffold incorporating such loaded particles will also be considered. This system can serve as a reservoir for nanoparticles allowing a controlled and sustained dosage for an extended period. Specifically, polylactide (PLA) has been selected as the biocompatible polymer matrix due to its wide applications in the biomedical field and even for its relatively low degradation rate. Porous scaffolds have been prepared by electrospinning since it is a technique that allows easy incorporation of nanoparticles, being also possible to minimize negative morphological changes (e.g., irregularities and formation of clumps) by accurate control of the processing parameters.

## 2. Results and Discussion

### 2.1. Encapsulation of CAM in ACP and cHAp Nanoparticles

**Figure 2:**
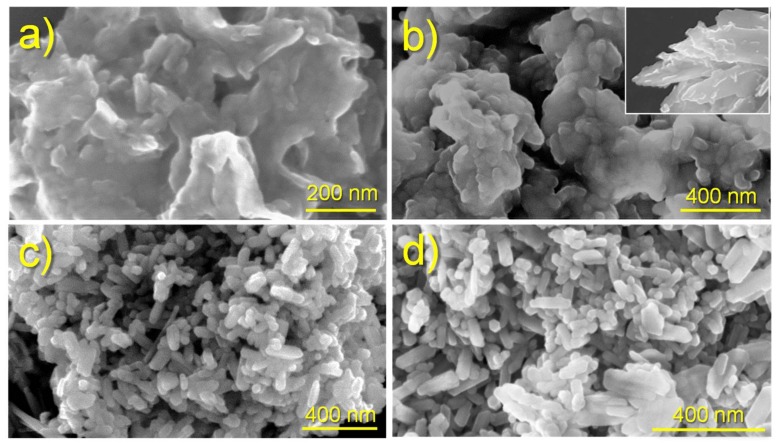
SEM images showing the morphology of nanoparticles of: (**a**) ACP, (**b**) ACP loaded with CAM (ACP-CAM), (**c**) cHAp and (**d**) cHAp loaded with CAM (cHAp-CAM). Inset of (**b**) shows typical CAM crystals.

Figure 2 shows the morphologies of ACP and cHAp particles prepared by precipitation of an ethanol solution of $\mathrm{Ca}{\left({\mathrm{NO}}_{3}\right)}_{2}\;$ with an aqueous solution of $\;{\left({\mathrm{NH}}_{4}\right)}_{2}{\mathrm{HPO}}_{4}\;$ and after the subsequent aging or hydrothermal treatment. In order to get appropriated rounded or rod-like morphologies that could be considered appropriate for drug encapsulation, the solutions containing ${\mathrm{Ca}}^{2+\;}$ and ${\mathrm{PO}}_{4}^{3-}$ ions were quickly mixed at pH 11, where hydroxyapatite is known to be stable [25,26,27]. The reaction mixture had a relatively high ethanol content to allow the addition of a subsequent ethanol solution of CAM without causing the precipitation/crystallization of this low water-soluble drug. The applied encapsulation methodology was focused to incorporate CAM once a former hydroxyapatite nucleus was obtained. Therefore, CAM was mainly loaded in the outer shell of nanoparticles in order to facilitate its release.

Significant morphological differences were found between the nanoparticles of ACP and cHAp. The first ones were rounded with a diameter around 50–70 nm, whereas cHAp crystallized as nanorods with a length of 200 nm and a width of around 90 nm. The degree of agglomeration of particles was also different, being higher for the amorphous ACP. Figure 2 also shows that the encapsulation of CAM had not a significant influence on the derived morphologies. Furthermore, no evidence of CAM crystals, which usually appear as platelets (inset of Figure 2b), was detected in the different preparations, as could be expected due to the established washing protocol.

**Figure 3:**
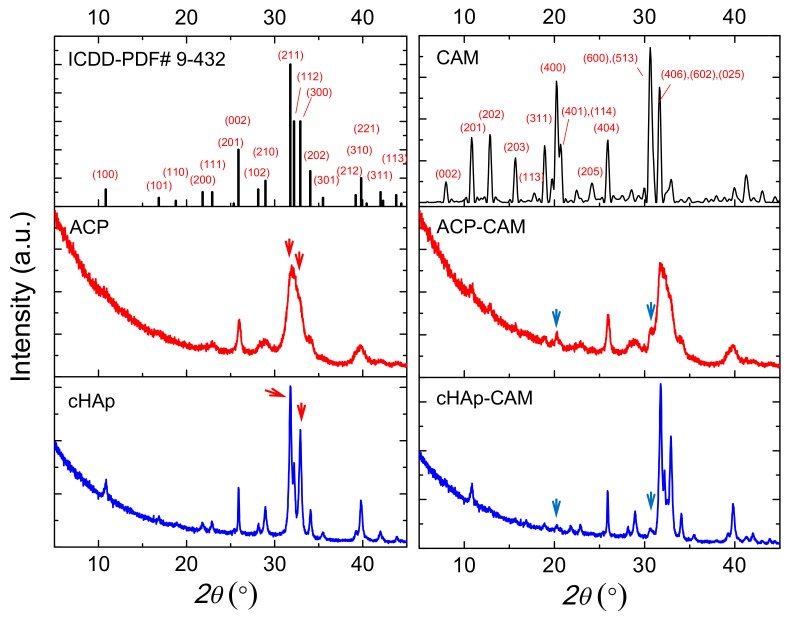
X-ray diffraction patterns of the reported diffraction pattern of hydroxyapatite (ICDD number 9-432), CAM, ACP nanoparticles, ACP-CAM nanoparticles, cHAp nanoparticles, and cHAp-CAM nanoparticles. Blue arrows point out the (401) + (114) and (600) + (513) reflections of CAM, while red arrows indicated the (211) and (300) reflections of ACP and cHAp.

As shown in Figure 3, ACP and cHAp particles have similar diffraction peaks, which correspond to the reported diffraction pattern of hydroxyapatite (ICDD number 9-432). The presence of a significant amount of impurities can, therefore, be discarded. X-ray diffraction patterns of ACP and cHAp particles showed clear differences that mainly concern the width of the reflection peaks, which obviously was greater for the worse crystallized ACP sample (Figure 3). For a more intuitive comparison of the differences in the nanostructure of the ACP and cHAp samples, the crystallite size was calculated through the Scherrer equation by measuring the full width at half maximum (FWHM). Results indicate that the crystallite size in the ACP sample was smaller than in the cHAp sample. This methodology has usually been employed for the study of crystallite size in differently doped calcium phosphates [28,29]. Degree of crystallinity was calculated considering the intensity of the (300) reflection, I_300_, and the intensity of the hollow between the (112) and (300) reflections, V_112/300_ (Equation (1)) [30]:

(1) $$\chi_{c}=1-\frac{V_{112/300}}{I_{300}}$$

Crystallinity values of 66% and 10% were determined for cHAp and ACP nanoparticles, respectively, while a slight decrease up to 55% and 9% was observed after the encapsulation of CAM. Distinctive peaks associated with CAM were difficult to be observed due to the small drug load. Thus, only a small signal at 0.428 nm, which corresponds to the (401) and (114) reflections of the CAM structure, could be intuited [31].

**Figure 4:**
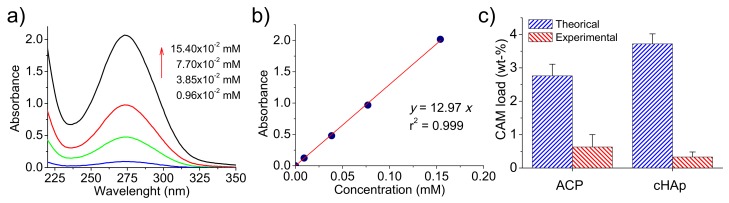
(**a**) UV–spectra of CAM solutions at the indicated concentrations. The spectra showed the absorption band of the *p*-nitrophenyl chromophore. (**b**) Calibration curve for absorbance measurements at 278 nm in PBS-EtOH medium. (**c**) Encapsulation efficiency for ACP and cHAp nanoparticles.

UV-spectra of CAM were recorded in the medium employed to dissolve the different kinds of nanoparticles and showed a characteristic band at 278 nm (Figure 4a). This well-known absorption band is attributed to the p-nitrophenyl chromophore group and has a clear relationship with CAM concentration in the medium, as can be observed in Figure 4b. Specifically, a calibration equation (y = 12.97x) with a linear regression coefficient (r ) of 0.999 was deduced as well as a molar extinction coefficient of 12970 $M^{-1}{\mathrm{cm}}^{-1}$.

The amount of encapsulated drug was, therefore, evaluated by UV measurements, with determined encapsulation efficiencies of 23% and 9% for ACP and cHAp, respectively. It is clear that great differences were found depending on the applied aging and hydrothermal treatments. Thus, CAM was more easily excluded from the crystalline domains, and consequently, a lower encapsulating efficiency was detected for these more crystalline particles. Alternatively, a certain amount of CAM could be released from the formed nanoparticles during the prolonged hydrothermal treatment at relatively high temperatures.

The amount of CAM loaded in the particles can also be referred to as a weight percentage. Values of 0.63% and 0.33% were determined for ACP and cHAp, respectively, as shown in Figure 4c where maximum percentages are also reported. These theoretical percentages (CAM_T_) are determined as (Equation (2)):

CAM_T_ (%) = m_CAM_/m_nan_ × 100 (2)

where m_CAM_ and m_nan_ are the weights of chloramphenicol added in the reaction medium and synthesized nanoparticles (ACP or cHAp), respectively.

Note that in this case, the maximum load is higher for cHAp due to its lower synthesis yield after the more drastic conditions of the hydrothermal treatment.

**Figure 5:**
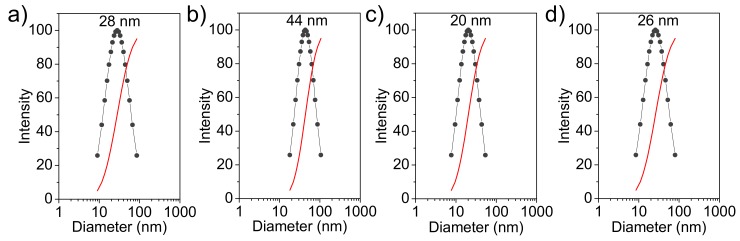
Nanoparticle diameter distribution determined by DLS for: (**a**) ACP, (**b**) ACP-CAM, (**c**) cHAp and (**d**) cHAp-CAM. Size distribution (black line) and measure correlation functions (red line).

Figure 5 shows the hydrodynamic diameter distribution (measured in water) of the prepared nanoparticles. This distribution is narrow (i.e., between 10 and 100 nm) and fits a unimodal Gaussian curve. Particles of ACP and cHAp showed very similar diameters with average values of 28 ± 8 nm and 20 ± 5 nm, respectively. The encapsulation of CAM increased the particle size and average values of 44 ± 10 nm and 26 ± 7 nm were determined. Thus, amorphous ACP had a higher increase (i.e., 57% with respect to 30% for cHAp), which is logical considering its higher CAM load.

**Figure 6:**
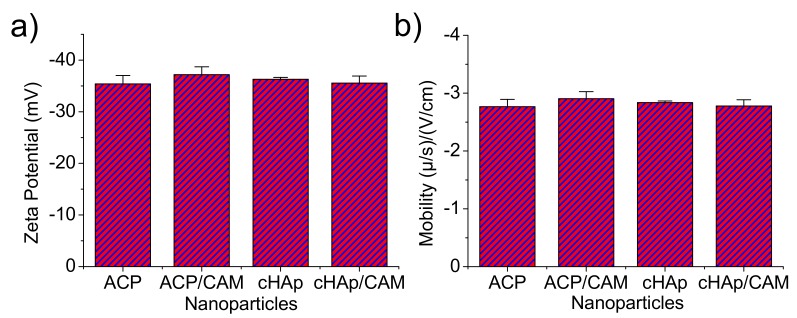
Zeta potential (**a**) and electrophoretic mobility (**b**) of the indicated unloaded and CAM loaded nanoparticles.

Z-potential and the electrophoretic mobility were also determined for the aqueous (pH 6.5) nanoparticle dispersions (Figure 6). It was clearly observed that the surface charge and electrophoretic mobility of the nanoparticles of ACP and cHAp with and without charge of CAM were similar and within the range of −35 to −40 mV and −2.7 to −3 (μ/s)/(V/cm), respectively. The similar potential Z values determined for the various particles supports the idea that CAM was only incorporated in the nanoparticles bulk since similar surface chemistry was derived. This fact was possible since the repetitive washing performed after the CAM encapsulation process allowed removing the potentially adsorbed molecules in the particle surface.

### 2.2. CAM Release from ACP and cHAp Nanoparticles

**Figure 7:**
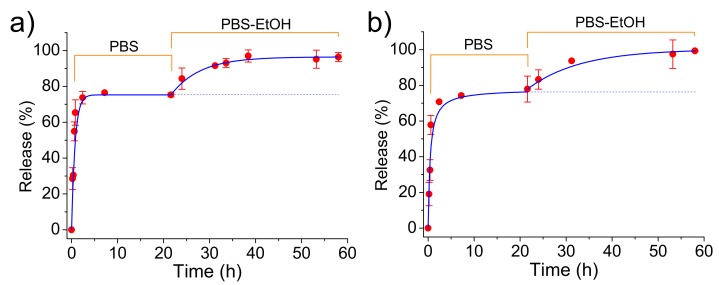
Release of CAM from ACP (**a**) and cHAp (**b**) loaded nanoparticles during exposure to the indicated media. PBS-EtOH refers to the PBS-ethanol mixture (see Methods Section).

CAM was easily released from both loaded ACP and cHAp nanoparticles in the simple physiological PBS medium. Figure 7 shows that CAM was released in a similar way from both types of nanoparticles although the loading efficiency and the antibacterial effect (as explained in the next section) were different. A fast delivery step that corresponded to the superficially located drug was first detected. The released percentages at the end of this step (up to 2 h) corresponded to about 58% for both types of nanoparticles. After that, the release mechanism changed to a first-order kinetic model that is indicative of a process where the rate of release is directly proportional to the drug concentration. Kinetic constant ($k_{1}=$ 0.30 and 0.23 $h^{-1}$ for ACP and cHAp loaded with CAM, respectively) was different for both types of nanoparticles, reflecting the significance on the release of the crystalline structure. The released percentages achieved a constant value close to 75% after 20 h. This stationary level was a main consequence of the saturation of the aqueous solution with the scarcely soluble CAM drug.

The release medium was subsequently changed by a PBS-ethanol mixture in order to get comparative data with the subsequent experiments performed with PLA scaffolds. Basically, in the last case, ethanol had a great influence on the release behavior due to its capacity to swell microfibers and facilitate the drug delivery. Figure 7 clearly shows that the release from both types of nanoparticles was again significant due to both the renovation of the medium and the higher solubility of CAM in ethanol. Thus, a complete delivery of CAM was achieved after an additional period of approximately 20 h. This release could be fitted with a first-order kinetic model with a higher constant than that previously determined. This constant decreased in a similar way as a consequence of the low CAM residual content for ACP and cHAp up to a value close to 0.12 and 0.07 $h^{-1}$, respectively.

### 2.3. Antibacterial Effect of Encapsulated CAM in ACP and cHAp Nanoparticles

**Figure 8:**
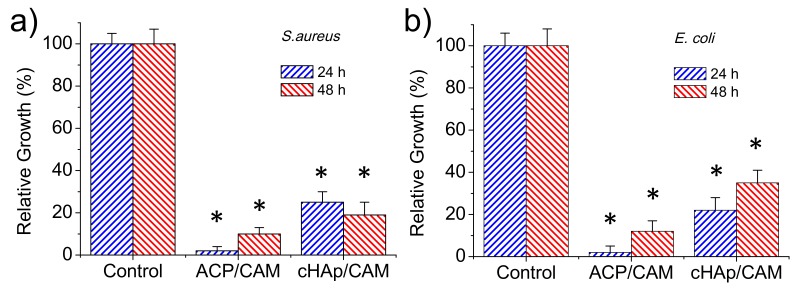
Relative growth of *S. aureus* (**a**) and *E. coli* (**b**) bacteria in the control and CAM loaded ACP and cHAp nanoparticles. * *p* < 0.05 vs. control.

The antibacterial effect of CAM-loaded nanoparticles was evaluated quantitatively by considering the growth curves of Gram-positive (*S. aureus*) and Gram-negative (*E. coli*) bacteria (Figure 8a,b, respectively). Results clearly pointed out that an antibacterial effect was preserved although CAM was effectively encapsulated in the different nanoparticles. This antibacterial effect was clearly higher for the ACP amorphous nanoparticles, with the bacterial growth reduced to an average value of 2% for both bacteria after 24 h as a consequence of the indicated fast release. Nevertheless, bacterial growth was possible at higher exposure times due to the extinction of CAM in the culture media once it was delivered. Thus, bacterial growths of 10% and 11% were determined after 48 h for *S. aureus* and *E. coli*, respectively. The lower CAM load of cHAp nanoparticles led to a lower antibacterial effect, with growth percentages of 25% and 21% found after 24 h for *S. aureus* and *E. coli*, respectively. The low release rate of CAM was demonstrated by the experiments performed with *S. aureus* since bacterial growth still decreased to 19% after 48 h of exposure. This effect was not clear for the *E. coli* bacterium since after this period the bacterial growth still increased (i.e., 34%).

**Figure 9:**
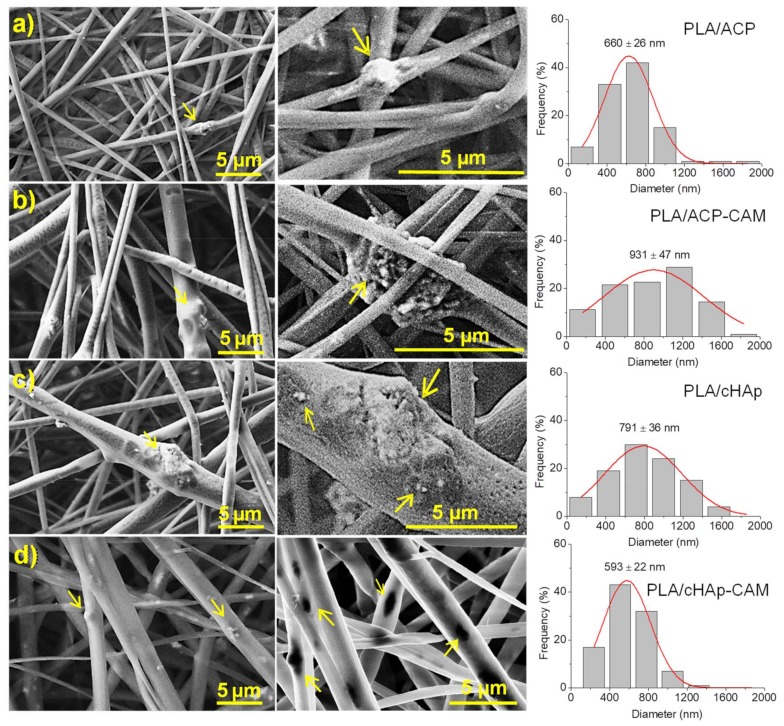
SEM images showing the morphology of PLA microfibers loaded with: (**a**) ACP, (**b**) ACP-CAM, (**c**) cHAp and (**d**) cHAp-CAM. Arrows point out the presence of nanoparticle agglomerates inside microfibers. In the middle column, higher magnification images are shown with aggregates of the nanoparticles inside the fibers. Image registered with the InLens detector (**d**, middle) that evidences the presence of nanoparticles as black spots (in this case the different phases of PLA matrix and the cHAp particles can be differentiated). The last column shows the distribution of the diameter of the fibers adjusted to a Gaussian model with the mean ± SE values.

Conditions for the electrospinning of PLA have been optimized in earlier works [32,33,34]. Thus, a chloroform-acetone mixture (2:1 v/v) with a polymer concentration of 8 wt-% was adequate to get fibers with diameters in the nano/micrometric range. Specifically, long continuous fibers with a narrow unimodal distribution of diameters (average size of 580 ± 10 nm) were obtained using operational parameters of 15 kV, 10 mL·$h^{-1}$ and 12 cm for the applied voltage, the flow rate, and the tip-collector distance, respectively. The same conditions could be successfully applied to get fibers loaded with medicated and non-medicated ACP and cHAp nanoparticles. Fibers were still continuous and relatively uniform (Figure 9), although some minor aggregates/crystallites could be distinguished near their surface, especially for the thickest ones (see arrows in Figure 9). In general, the incorporation of nanoparticles led to a higher diameter dispersion (an even a slight increase of the average diameter), probably as a consequence of the protuberances caused by the incorporated particles. The conductivity of polymer solutions could also be slightly changed by the addition of nanoparticles, but probably its influence on the electrospinning process would be scarce. Results show also that the encapsulation of CAM has not a clear effect on the final morphology if the size of particles is not considered. However, it should be pointed out that larger aggregates and smaller nanorods were obtained after the encapsulation of CAM as shown in Figure 2. Thus, fibers loaded with ACP or cHAp showed an increase and a decrease of the average diameter from 660 to 930 nm and from 790 to 590 nm, respectively, when CAM was encapsulated.

### 2.4. Preparation of Electrospun PLA Microfibers Incorporating ACP and cHAp Nanoparticles with or without Encapsulated CAM 

**Figure 10:**
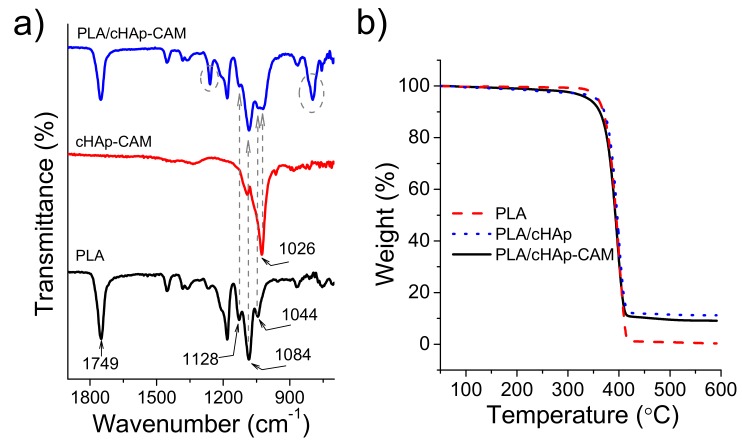
(**a**) FTIR spectra for PLA (black), cHAp-CAM (red) and the PLA scaffold incorporating cHAp nanoparticles encapsulating CAM (blue). (**b**) Thermogravimetric curves of PLA (red), PLA microfibers incorporating cHAp (blue) and PLA microfibers incorporating CAM loaded cHAp nanoparticles (black).

FTIR spectra of PLA electrospun fibers incorporating hydroxyapatite nanoparticles were practically identical to the spectrum of raw PLA even when CAM was encapsulated (Figure 10a). Thus, typical bands of the drug (e.g., vibrational peaks of the carbonyl group (C=O ), C=C stretching and ${\mathrm{NO}}_{2}$ stretching at 1694, 1560 and 1512 ${\mathrm{cm}}^{-1}$, respectively) were never detected as presumed from the low CAM load. The higher content in nanoparticles gave rise to a minor change in the spectrum that logically was seen around 1026 ${\mathrm{cm}}^{-1}$ where hydroxyapatite shows the most intense signal [29]. Specifically, the low intense PLA band at 1044 ${\mathrm{cm}}^{-1}\;$ appeared overlapped with the indicated peak for both ACP and cHAp loaded samples. Therefore, this unequivocal trace of the calcium phosphate salt demonstrated that at least a small part of hydroxyapatite remained close to the fiber surface. Thermogravimetric curves (Figure 10b) showed that the thermal stability was not influenced by the incorporation of any kind of medicated hydroxyapatite. Thus, PLA always decomposed according to a single step with a maximum weight loss rate of around 405 °C. Obviously, the char yield measured at 500 °C was in full agreement with the load amount of nanoparticles (i.e., 0.5% and 10% for PLA and PLA loaded with ACP or cHAp, respectively). Samples loaded with nanoparticles, and independently of the presence of encapsulated CAM, showed a small and continuous weight loss from 100 °C to 300 °C, which could be associated to the presence of volatile compounds introduced during the synthesis of nanoparticles. The onset degradation temperature of PLA was never affected, a feature that is significant since it indicates that processing was not influenced by the presence of CAM molecules if they were properly encapsulated in the ACP or cHAp nanoparticles.

All prepared PLA scaffolds had a hydrophobic surface independently of incorporating unloaded and CAM loaded nanoparticles and independently of the type/crystallinity of hydroxyapatite nanoparticles. Thus, contact angle measurements (not shown) gave always values in the 125°–128° range.

### 2.5. CAM Release from PLA Electrospun Scaffolds Incorporating ACP and cHAp Nanoparticles with Encapsulated CAM 

**Figure 11:**
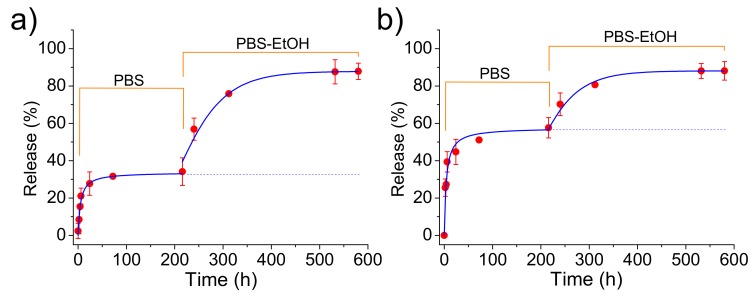
Release of CAM from PLA electrospun microfibers loaded with ACP-CAM (**a**) and cHAp-CAM nanoparticles (**b**) during exposure to the indicated media. PBS-EtOH refers to the PBS-ethanol mixture (see Methods Section).

Release measurements in the PBS medium indicated that CAM was effectively delivered (Figure 11) despite the loaded ACP or cHAp particles were embedded in the PLA microfibers. Logically, the delivery was clearly delayed with respect to that observed from the nanoparticles alone since CAM had to diffuse through the polymer matrix. Specifically, first-order kinetic constants of 0.17 and 0.19 $h^{-1}\;$ were calculated for microfibers incorporating ACP and cHAp particles, respectively. In any case, release percentages of 55% and 32% were determined after 20 h of exposure, justifying the use of this kind of scaffolds for the slow delivery of CAM. Differences on the release should be justified, in this case, as a consequence of the lower CAM load for PLA incorporating cHAp that reduces the driving force for the diffusion process. Experimental data show that an enhanced diffusion caused by the thinner fiber thickness in the scaffold cannot compensate for the effect derived from the low drug loading rate in cHAp. The influence of scaffold morphology differences can be considered negligible since a contrary behavior could be expected for the slightly thinner fibers obtained when cHAp was incorporated. Note also that the release achieved again a stable value but at percentages clearly lower than observed for the release from the nanoparticles alone. We interpret that, in this case, this constant level is not a consequence of the limited solubility of CAM in PBS since the previously determined percentage should be expected. Therefore, the observed decrease in the release percentage seems to indicate the presence of encapsulated CAM in the inner particles (i.e., distant from the fiber surface).

The addition of ethanol in the release medium made feasible the access of solvent to these inner particles. Thus, complete delivery was observed after a period of 40 h. Kinetics obeyed again a first-order equation, being $k_{1}$ dependent of the type of nanoparticle due to the different CAM load and even on the different facility to release the encapsulated compound. Thus, values of 0.30 and 0.21 ${\mathrm{cm}}^{-1}\;$ were calculated for samples containing ACP and cHAp particles, respectively. In summary, ethanol made effective the release due to its great affinity with CAM and also to its capability to swell the PLA microfibers and facilitate the diffusion of CAM molecules through the polymer matrix.

### 2.6. Bacteriostatic Effect of PLA Scaffolds Incorporating ACP and cHAp Nanoparticles with Encapsulated CAM 

**Figure 12:**
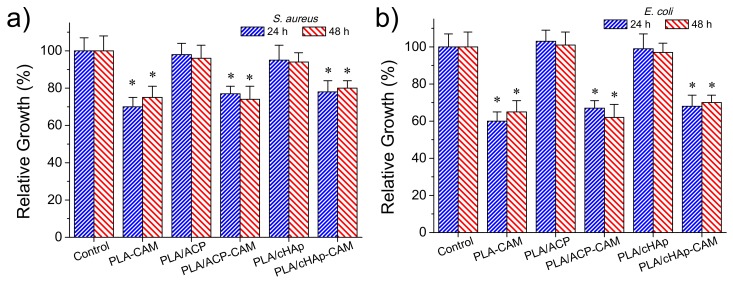
Relative growth of *E. coli* (**a**) and *S. aureus* (**b**) bacteria in the control and the indicated PLA scaffolds, * *p* < 0.05 vs. control.

The antimicrobial effect of CAM loaded matrices was again evaluated quantitatively by considering the growth inhibition of Gram-negative (*E. coli*) and Gram-positive (*S. aureus*) bacteria (Figure 12a,b, respectively). The control and the unloaded PLA/ACP and PLA/cHAp matrices were highly susceptible to bacterial infection. On the contrary, the relative bacterial growth clearly diminished when the PLA matrix was directly loaded with the drug as well as when PLA was loaded with the drug previously encapsulated in the hydroxyapatite nanoparticles. In the three cases, the relative growth diminished to values between 60% and 70%, demonstrating the diffusion of CAM through the PLA microfibers and sufficient release in the culture medium to render a bacteriostatic effect.

No significant differences were found after 24 and 48 h of culture suggesting a continuous release of the drug from the matrix, a feature that is in agreement with the results given in the previous section. Differences between the three loaded matrices were not highly significant (e.g., the relative growth of *E. coli* bacterium diminished to 60%–64%, 66%–62% and 68%–70% for PLA/CAM, PLA/ACP-CAM, and PLA/cHAp-CAM, respectively). Nevertheless, a slightly highest effectivity was detected when CAM was directly loaded in the microfibers and a slightly lower activity when CAM was encapsulated in cHAp. It should be pointed out that this small difference demonstrated that the activity could be well preserved despite both the significant decrease of the amount of loaded drug and the greater difficulty in releasing the encapsulated drug. In any case, the observed growth percentages from the studied PLA matrices were logically higher than observed from the loaded nanoparticles (e.g., 60%–70% in form of ca. 20%) (Figure 8), demonstrating certain difficulty of the drug to diffuse through the PLA nanofiber. Similar conclusions could be inferred when the activity of CAM loaded PLA matrices against *S. aureus* bacterium was evaluated (Figure 12b). However, in this case, the relative bacterial growth was slightly higher (e.g., 70%–80% with respect to 60%–70%) as a consequence of the lower susceptibility of *S. aureus* bacterium towards CAM. In addition, the differences between the different PLA loaded scaffolds were even less significant than detected for the *E. coli* bacterium.

### 2.7. Cytotoxicity of PLA Scaffolds Incorporating ACP and cHAp Nanoparticles with Encapsulated CAM 

**Figure 13:**
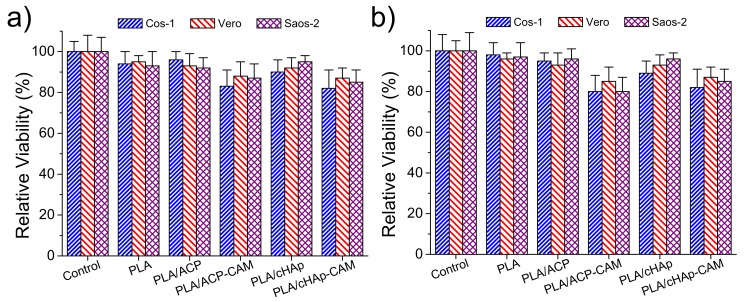
Relative viability for adhesion (**a**) and proliferation experiments for COS-1 (blue), VERO (red) and SAOS-2 (lilac) cells in the control and the indicated scaffolds. Significant differences (*p* < 0.05 level) were not observed from statistical analyses.

Adhesion and proliferation assays (Figure 13) demonstrated that PLA scaffolds incorporating ACP and cHAp were biocompatible and non-cytotoxic. Thus, the observed relative viabilities of the three selected cell lines (i.e., COS-1, VERO, and SAOS-2) onto the selected scaffolds were practically identical to those determined for the control. On the contrary, slight toxicity was detected from CAM-loaded samples. In any case, the viability decreased only to a value close to 80% in both adhesion and proliferation studies. This toxicity was slightly more noticeable for PLA samples having ACP nanoparticles due to their greater encapsulation efficiency. Nevertheless, it should be pointed out that statistical analyses indicated that the observed differences between all studied samples were not significant as shown in Figure 13. It is clear that the amount of released drug in the short cell exposure time of the two assays was not enough to produce a relevant toxic effect.

## 3. Materials and Methods

### 3.1. Materials

Polylactide, a product of Natureworks^®^ (polymer 2002D), was kindly supplied by Nupik International (Polinyà, Spain). According to the manufacturer, this PLA has a D content of 4.25 wt-%, a residual monomer content of 0.3 wt-%, a relative density of 1.24 g/cc, a glass transition temperature (T_g_) of 58 °C and a melting point of 153 °C.

The microbial culture was prepared with reagents and labware from Scharlau. *Escherichia coli* and *Staphylococcus aureus* bacteria strains were obtained from the Spanish Collection of Type Culture (CECT, Valencia, Spain). Human osteoblast-like cells (SAOS-2) and African green monkey fibroblasts (COS-1) and epithelial cells (VERO) were obtained from American Type Culture Collection (ATCC) (LGC Standards S.L.U., Barcelona, Spain).

### 3.2. Synthesis of ACP and HAp Nanoparticles

An aqueous solution of diammonium hydrogen phosphate, (${\left({\mathrm{NH}}_{4}\right)}_{2}{\mathrm{HPO}}_{4}$; 750 mM) was dropwise added over an ethanol solution of calcium nitrate ($\mathrm{Ca}{\left({\mathrm{NO}}_{3}\right)}_{2}$; 750 mM) under low stirring (100 rpm). The pH of the final mixture was continuously adjusted to 11 using an aqueous ammonium hydroxide solution (28%–30%). The reagent concentrations were consistent with the required Ca/P ratio of 1.67. The mixture was maintained under agitation (ca. 250 rpm) during 1 h. ACP was obtained by a simple aging treatment (i.e., allowing samples to stand overnight at 37 °C). Samples were washed three times with Milli-Q water and recovered by centrifugation after each step. Finally, white powders were obtained by lyophilization of the frozen samples (−80 °C). cHAp was prepared by applying a hydrothermal treatment instead of the indicated aging process. To this end, samples were loaded in a pressure vessel at 150 °C and 200 bars for 24 h. After this, a white powdered cHAp was recovered by centrifugation and washed three times with Milli-Q water. cHAp powder was obtained after drying by lyophilization.

### 3.3. Encapsulation of CAM in ACP or cHAp Nanoparticles

A 154 mM solution of CAM in absolute ethanol was immediately added after initiating the precipitation process of the ethanol solution of calcium nitrate with aqueous diammonium hydrogen phosphate. The added volume was calculated to get a 4.3 mM concentration of CAM in the final mixture. Aging and hydrothermal treatments to get encapsulated amorphous (ACP-CAM) and crystalline (cHAp-CAM) samples were performed according to the indicated protocols.

Encapsulation efficiency (EE% ) was calculated as Equation (3):

(3) $$EE\%=\left(\frac{W_{t}}{W_{0}}\right)\cdot 100$$

where $W_{t}\;$ is the total amount of the incorporated CAM and $W_{0}$ is the total quantity of CAM that was initially added in the precipitation medium. $W_{t}\;$ and $W_{0}\;$ were determined from UV measurements using an UV-Vis/NIR 3600 spectrometer (Shimadzu, Japan). To this end, linear calibration curves were obtained by plotting the absorbance measured at 278 nm versus CAM concentration prepared in PBS (phosphate buffer saline) supplemented with 70%-*v*/*v* of ethanol (PBS-EtOH). To quantify encapsulated CAM, the nanoparticles (20 mg) were dissolved with 200 µL of 100 mM HCl  and 50 mM NaCl mixture. The CAM was extracted with 1 mL of PBS-EtOH and quantified by UV-Vis spectroscopy.

### 3.4. Electrospinning of PLA Incorporating ACP or cHAp Nanoparticles

PLA was dissolved in a chloroform–acetone mixture (2:1 *v*/*v*) at a concentration of 8 *w*/*v*. Electrospun fibers were collected on a target placed at 12 cm from the needle tip (inside diameter 0.84 mm). The voltage was 15 kV and applied to the target using a high-voltage supply (Gamma High Voltage Research, ES30-5W). Polymer solutions were delivered via a single KDS100 infusion syringe pump (KD Scientific Inc., Holliston, MA, USA) at a flow rate of 10 mL·h^−1^. All electrospinning experiments were carried out at room temperature. ACP or cHAp (nanoparticles with and without incorporation of CAM) loaded electrospun fibers were prepared using the same indicated conditions. The nanoparticles content in the electrospinning solution was 1.6 *w*/*v*-% and 20 *w*/*w*-% for the electrospun scaffold.

### 3.5. Measurements

#### 3.5.1. Fourier Transform Infrared (FTIR) Spectroscopy 

Infrared absorption spectra were recorded in the 4000–600 ${\mathrm{cm}}^{-1}\;$ range with a Fourier transform FTIR 4100 Jasco spectrometer equipped with a Specac model MKII Golden Gate attenuated total reflection (ATR) cell.

#### 3.5.2. Thermal Stability 

Thermogravimetric analyses (TGA) for studying thermal stability at relatively low temperatures (<600 °C) were performed at a heating rate of 20 °C/min (sample weight ca. 5 mg) with a Q50 thermogravimetric analyzer of TA Instruments and under a flow of dry nitrogen. Test temperatures ranged from 50 to 600 °C.

#### 3.5.3. Wettability 

Contact angles (CA) were measured at room temperature with sessile drops using an OCA-15 plus contact angle microscope (Dataphysics Instruments GmbH, Fiderstadt, Germany) and SCA20 software. Contact angle values of the right and left sides of distilled water drops were measured and averaged. Measurements were performed 10 s after the drop (5 µL) was deposited on the sample surface. All CA data were an average of six measurements on different surface locations.

#### 3.5.4. Hydrodynamic Size 

Dynamic light scattering (DLS) studies were performed using NanoBrook Omni Zeta Potential Analyzer (Brookheaven Instruments Co., Blue Point Road Holtsville, NY, USA). Samples were resuspended in Milli-Q water at a concentration of 2 *w*/*v*-% and placed into a cuvette of polystyrene with light pass of 1 cm to be analyzed at 25 °C using a scattering angle of 90°. Measurement consisted of three runs each of 120 s duration and averaged to obtain the effective diameter ($D_{eff}$ ).

#### 3.5.5. Zeta Potential 

Z-potential measurements, based on Doppler velocimetry (electrophoretic light scattering, ELS), were performed using NanoBrook Omni Zeta Potential Analyzer (Brookheaven Instruments Co., Blue Point Road Holtsville, NY, USA).The particles were resuspended in 1 mM KCl solution to obtain a dilute suspension of 1 × 10^−3^ *w*/*v*-% and 30 consecutive measurements were taken of each sample and averaged to obtain the Z-potential.

#### 3.5.6. X-ray Diffraction 

X-ray diffraction patterns were acquired using a Bruker D8 Advance model with ${\mathrm{CuK}}_{\alpha }$ radiation (λ = 0.1542 nm), Bragg–Bretano, θ–2θ geometry and one-dimensional Lynx Eye detector. Diffraction profiles were processed using PeakFit v4 software (Jandel Scientific Software) and the graphical representation performed with OriginPro v8 software (OriginLab Corporation, Northampton, MA, USA).

#### 3.5.7. Scanning Electron Microscopy (SEM) 

Detailed inspection of texture and morphology of electrospun samples was conducted by scanning electron microscopy using a Focused Ion Beam Zeiss Neon 40 instrument (Carl Zeiss, Oberkochen, Germany). Carbon coating was accomplished by using a Mitek K950 Sputter Coater fitted with a k150× film thickness monitor. Samples were visualized at an accelerating voltage of 5 kV. The diameter of electrospun fibers was measured with SmartTiff software from Carl Zeiss SMT Ltd.

### 3.6. Release Experiments

Controlled release measurements were performed with 4 mg of loaded nanoparticles of ACP and cHAp as well as with 0.5 × 0.5 cm square pieces of the different loaded scaffolds (i.e., those with CAM encapsulated in ACP or cHAp). The thickness of these scaffolds was always close to 100 µm. The corresponding samples were incubated at 37 °C in an orbital shaker at 80 rpm in tubes of 10 mL. Phosphate-buffered saline (PBS) and a 3:7 *v*/*v* mixture of PBS and ethanol were employed as release media following a two-step process. This consisted of a first release in the PBS medium during 22 h, which was the time necessary to get a constant value, and a second step for the next 40 h in the PBS-ethanol medium. Drug concentration was evaluated by UV absorbance measurements as described above but using linear calibration curves of the corresponding release media. All drug release tests were carried out using three replicates and the results obtained were averaged. The combination of the Higuchi [35] and first-order [36] models was employed to describe the first (0%–60%) and last part of the release (40%–100%), respectively.

### 3.7. Antibacterial Test Assays

*E. coli* and *S. aureus* bacteria were selected to evaluate the antibacterial effect of CAM-loaded nanoparticles and the derived electrospun scaffolds. The bacteria were previously grown aerobically to exponential phase in broth culture (5 g/L beef extract, 5 g/L NaCl, 10 g/L tryptone, pH 7.2).

Growth experiments were performed on a 24-well culture plate. CAM loaded ACP and cHAp nanoparticles (20 mg) and differently loaded electrospun PLA scaffolds (pieces of 1 × 1 cm and thickness close to 100 µm) were placed into the wells. Unloaded ACP and cHAp nanoparticles and PLA scaffolds were considered as negative controls, and PLA loaded with CAM was a positive control. Then, 2 mL of broth culture containing 103 colony forming units (CFU) was added to the samples. Cultures were incubated at 37 °C and shakered at 80 rpm. Aliquots of 100 μL were taken at 24 h and 48 h from the starting culture time for absorbance measurement at 600 nm in a microplate reader. Thus, turbidity was directly related to the relative bacterial growth by considering the maximum growth attained in the absence of any polymeric matrix (control). Experiments were performed in quadruplicate and the results averaged.

### 3.8. Cell Adhesion and Proliferation Studies

Studies were performed with osteoblast SAOS-2 cells, fibroblast COS-1 cells and epithelial VERO cells. In all cases, cells were cultured in Dulbecco’s modified Eagle medium (DMEM), as previously reported [37].

Five pieces (1 cm × 1 cm and thickness close to 700 µm) of unloaded and CAM loaded scaffolds were placed and fixed in each well of a 24-well culture plate with a small drop of silicone (Silbione^®^ MED ADH 4300 RTV, Bluestar Silicones France SAS, Lyon, France). This plate was then sterilized by UV-radiation in a laminar flux cabinet for 15 min. For cell adhesion assays, aliquots of 50–100 μL containing 2 × $10^{5}$ cells, were seeded onto the samples in each well and incubated for 24 h (adhesion assay). For cell proliferation assays, the same aliquot volume but containing a lower cell concentration than for adhesion experiments (1 × $10^{5}\;$ cells) was seeded and incubated for 96 h.

Samples were evaluated by the standard adhesion and proliferation method [35]. The procedure is based on a simple modification of the ISO10993–5:2009 standard test, which describes the appropriate methodology to assess in vitro cytotoxicity of medical devices. This test is designed to determine the in vitro biological response of mammalian cells using appropriate biological parameters. According to this ISO standard, devices fall into one of three categories based on expected contact with the patient: (a) Limited (≤24 h), (b) prolonged (>24 h and ≤30 days), and (c) permanent (>30 days). In our case, the assay was performed according to the limited and prolonged categories and using four replicates. The results were then averaged.

### 3.9. Statistical Analysis

The statistical analysis of the data was performed with OriginPro v10 software. The data were statistically analyzed using one-way analysis of variance (one-way ANOVA). Multiple comparisons were carried out using the Tukey test with statistical significance at *p* < 0.05.

## 4. Conclusions

CAM can be encapsulated within both amorphous and crystalline hydroxyapatite nanoparticles, with differences in the encapsulation efficiency according to the crystallinity of the sample. The selected encapsulation process allows incorporating CAM once nuclei of hydroxyapatite were formed, and consequently, CAM should be mainly loaded in the nanoparticle shell. In this way, the drug could be easily delivered even in an aqueous medium where it was scarcely soluble. This fast release conferred a clear antibacterial character to both types of nanoparticles. The aging and hydrothermal treatment performed with the precipitated nanoparticles had logically a great influence on the sample crystallinity but also on the encapsulation efficiency.

CAM encapsulated hydroxyapatite nanoparticles could be easily incorporated into polylactide microfibers by means of the electrospinning technique. The obtained fibers were continuous and relatively uniform in size but showed irregularities as a consequence of the presence of some nanoparticle agglomerates. CAM release in an aqueous PBS medium from such fibers was hindered with regard to that observed from nanoparticles. Nevertheless, a small CAM fraction could be faster released, conferring to the scaffold a bacteriostatic character. Scaffolds were biocompatible due to the low release rate of CAM. A significant drug percentage remained in the nanoparticles, making the scaffold interesting as a CAM reservoir.
